# QClus: a droplet filtering algorithm for enhanced snRNA-seq data quality in challenging samples

**DOI:** 10.1093/nar/gkae1145

**Published:** 2024-12-05

**Authors:** Eloi Schmauch, Johannes Ojanen, Kyriakitsa Galani, Juho Jalkanen, Kristiina Harju, Maija Hollmén, Hannu Kokki, Jarmo Gunn, Jari Halonen, Juha Hartikainen, Tuomas Kiviniemi, Pasi Tavi, Minna U Kaikkonen, Manolis Kellis, Suvi Linna-Kuosmanen

**Affiliations:** Computer Science and Artificial Intelligence Laboratory, Massachusetts Institute of Technology, 32 Vassar St, Cambridge, MA 02139, USA; Broad Institute of MIT and Harvard, 415 Main Street, Cambridge, MA 02142, USA; A. I. Virtanen Institute for Molecular Sciences, University of Eastern Finland, Neulaniementie 2, 70211 Kuopio, Finland; Computer Science and Artificial Intelligence Laboratory, Massachusetts Institute of Technology, 32 Vassar St, Cambridge, MA 02139, USA; Broad Institute of MIT and Harvard, 415 Main Street, Cambridge, MA 02142, USA; A. I. Virtanen Institute for Molecular Sciences, University of Eastern Finland, Neulaniementie 2, 70211 Kuopio, Finland; Computer Science and Artificial Intelligence Laboratory, Massachusetts Institute of Technology, 32 Vassar St, Cambridge, MA 02139, USA; Broad Institute of MIT and Harvard, 415 Main Street, Cambridge, MA 02142, USA; Medicity Research Laboratories, University of Turku, Tykistökatu 6, 20520 Turku, Finland; A. I. Virtanen Institute for Molecular Sciences, University of Eastern Finland, Neulaniementie 2, 70211 Kuopio, Finland; Medicity Research Laboratories, University of Turku, Tykistökatu 6, 20520 Turku, Finland; Department of Anesthesiology and Operative Services, Kuopio University Hospital, Puijonlaaksontie 2, 70029 KYS, Finland; Heart Center, Turku University Hospital, Hämeentie 11, 20521 Turku, Finland; Department of Medicine, University of Turku, Kiinamyllynkatu 10, 20520 Turku, Finland; Medical School, University of Eastern Finland, Yliopistonranta 8, 70210 Kuopio, Finland; Heart Center, Kuopio University Hospital, Puijonlaaksontie 2, 70211 Kuopio, Finland; Medical School, University of Eastern Finland, Yliopistonranta 8, 70210 Kuopio, Finland; Heart Center, Kuopio University Hospital, Puijonlaaksontie 2, 70211 Kuopio, Finland; Heart Center, Turku University Hospital, Hämeentie 11, 20521 Turku, Finland; Department of Medicine, University of Turku, Kiinamyllynkatu 10, 20520 Turku, Finland; Cardiovascular Medicine and Network Medicine Division, Brigham and Women’s Hospital, Harvard Medical School, 75 Francis St, Boston, MA 02115, USA; A. I. Virtanen Institute for Molecular Sciences, University of Eastern Finland, Neulaniementie 2, 70211 Kuopio, Finland; A. I. Virtanen Institute for Molecular Sciences, University of Eastern Finland, Neulaniementie 2, 70211 Kuopio, Finland; Computer Science and Artificial Intelligence Laboratory, Massachusetts Institute of Technology, 32 Vassar St, Cambridge, MA 02139, USA; Broad Institute of MIT and Harvard, 415 Main Street, Cambridge, MA 02142, USA; Computer Science and Artificial Intelligence Laboratory, Massachusetts Institute of Technology, 32 Vassar St, Cambridge, MA 02139, USA; Broad Institute of MIT and Harvard, 415 Main Street, Cambridge, MA 02142, USA; A. I. Virtanen Institute for Molecular Sciences, University of Eastern Finland, Neulaniementie 2, 70211 Kuopio, Finland

## Abstract

Single-nuclei RNA sequencing remains a challenge for many human tissues, as incomplete removal of background signal masks cell-type-specific signals and interferes with downstream analyses. Here, we present Quality Clustering (QClus), a droplet filtering algorithm targeted toward challenging samples. QClus uses additional metrics, such as cell-type-specific marker gene expression, to cluster nuclei and filter empty and highly contaminated droplets, providing reliable filtering of samples with varying number of nuclei and contamination levels. In a benchmarking analysis against seven alternative methods across six datasets, consisting of 252 samples and over 1.9 million nuclei, QClus achieved the highest quality in the greatest number of samples over all evaluated quality metrics and recorded no processing failures, while robustly retaining numbers of nuclei within the expected range. QClus combines high quality, automation and robustness with flexibility and user-adjustability, catering to diverse experimental needs and datasets.

## Graphical Abstract

**Graphical Abstract:**
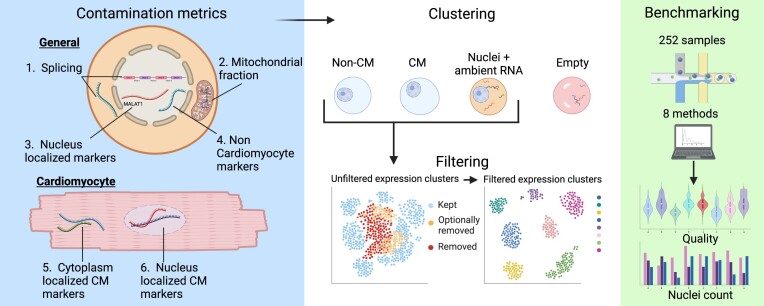

## Introduction

Single-cell RNA sequencing is a powerful tool for understanding the complex transcriptomes of heterogeneous cell populations (1,2). Single-nuclei RNA sequencing (snRNA-seq) uses the same principle, but isolates nuclei instead of cells (1). Both methods are growing in use and popularity in biomedical research (3); however, snRNA-seq is particularly well suited for solid and frozen tissues, as well as for more challenging tissues, such as the human heart. In these tissues, the whole cells may be difficult to isolate or the use of the single-cell approach may lead to biased cell yields due to the differences in cell sizes between the tissue cell populations [e.g., cardiomyocyte (CM) and non-cardiomyocyte (non-CM) populations in the heart] (4,5).

**Figure 1.**
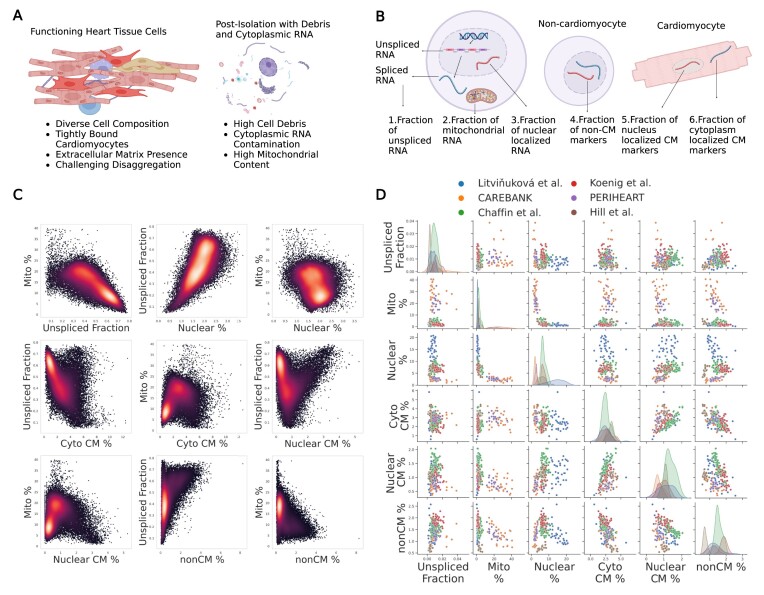
Modeling ambient RNA contamination using general as well as cell-type-specific quality metrics in human heart snRNA-seq data. (**A**) Tightly connected CMs in living human cardiac tissue (left panel) and nuclei (post isolation), containing significant amounts of debris and contamination (right panel). (**B**) General as well as cell-type-specific metrics for modeling contamination that is heavily contributed to by CMs. (**C**) Density-colored plots of the metrics comparisons for a single sample (CAREBANK, sample CB-S00). (**D**) Metrics across six datasets and 252 samples, showing high inter-sample and inter-dataset variability. Created in BioRender [Linna-kuosmanen, S. (2024); https://BioRender.com/j43n205].

Droplet-based snRNA-seq works by encapsulating single nuclei in droplets, where each droplet contains RNA from one nucleus. However, ambient RNA contamination, such as cytoplasmic or cell-free RNA from the input solution, can contaminate the droplets. This is a significant concern for solid tissues, leading to misidentification of cell types and states (6). For example, in the human heart, CMs, the contractile units of the heart, are tightly bound together (7), large in size, with high mitochondrial count, and they produce high amounts of transcripts due to their size and metabolic activity (8), which can lead to more cell debris and cytosolic RNA in the nuclei suspensions (Figure 1A) and the resulting droplets (8,9). This can result in mistaken interpretation of gene expression patterns (6), especially when CM transcripts are confused for genuine signals from other cell types. Such complications necessitate rigorous quality control in snRNA-seq workflows. In addition to heavily contaminated droplets, accurate exclusion of empty droplets is pivotal to avoid bias and ensure accuracy of the subsequent analyses (10).

Conventional methods for empty droplet removal in snRNA-seq often combine Unique Molecular Identifier (UMI) distribution filtering [generally included in preprocessing programs, such as 10X Genomics Cell Ranger (11)] and mitochondrial percentage-based filtering (6,10,12–15). These techniques rely on a clear distinction between empty and nuclei-containing droplets, which is typically true for samples with average contamination levels. However, in several tissue types, including the heart, elevated cytoplasmic contamination levels render standard removal methods inadequate (6). Recently, multiple snRNA-seq atlases have been shown to include clusters of low quality (defined by high splicing levels), demonstrating the need for improved quality control methods (16). Recognizing this, recent years have seen the advent of bioinformatics tools aimed at identifying empty and highly contaminated droplets for removal or modeling contamination and correcting the expression count matrix directly (6,10,17–19). Studies highlight the importance of splicing information (6) and nuclear gene expression (20), in addition to the previously established metric that is mitochondrial gene expression. This suggests that droplet filtering methods could use a combination of these metrics to remove empty and highly contaminated droplets.

In this study, we focused on determining the most effective method of droplet filtering in extensive datasets of challenging samples with minimal manual input, using human cardiac tissue data as an example. After reviewing current methods (6,9,10,17–19,21) and testing them on heart snRNA-seq data, we opted for a new approach to improve data quality by contamination-based droplet filtering. Our primary objective was to develop a method that reliably and automatically works for large datasets, even when such datasets demonstrate high inter-sample variability. We sought to construct a method that needed minimal manual input and no sample-level parameter tuning. To this end, we developed a quality clustering algorithm, QClus, an innovative droplet filtering method that employs unsupervised clustering of general as well as cell-type-specific quality metrics. QClus, when compared with standard practices and prior algorithms, showed enhanced results across samples. It was specifically designed for nuclei calling instead of count decontamination, as our goal was to ensure the accurate identification of individual nuclei in the dataset, rather than attempting to filter or adjust the expression counts of detected transcripts. By focusing on nuclei calling, we aimed to lay a foundation for subsequent analyses, ensuring that any interpretations or insights derived from the data would be based on correctly identified cellular components. By ensuring more dependable datasets, our methodology may help offer deeper insights into tissue biology.

## Materials and methods

### Datasets and preprocessing

For the four external datasets, the FASTQ files from each sample were downloaded from their respective repository. The Hill *et al.* (22) and Koenig *et al.* (23) data were obtained from GEO (respectively GSE203275 and GSE183852). The Litviňuková *et al.* (5) dataset was obtained from ENA (https://www.ebi.ac.uk/ena/browser/view/PRJEB39602). The Chaffin *et al.* (24) data were obtained from dbGaP (https://www.ncbi.nlm.nih.gov/projects/gap/cgi-bin/study.cgi?study_id=phs001539.v1.p1) with proper authorizations. The CAREBANK and PERIHEART data originate from Linna-Kuosmanen et al. (33) (https://doi.org/10.5281/zenodo.10822323).

More information about the datasets, their experimental processing and the patient’s conditions, can be found in their respective papers (5,22–24,33).

All samples from each dataset were processed in parallel using a dedicated high-performance computing cluster in our laboratory, managed via the SLURM workload manager.

Package versions were as follows:

- CellBender 0.2.2;
- Velocyto 0.17.17;
- Scanpy 1.9.2;
- SampleQC 0.6.6;
- DIEM 2.4.1;
- Celda 1.14.1 for the use of DecontX;
- DropletQC 0.0.0.9000; and
- EmptyNN 1.0.

Cell Ranger v7 (11) was run for all samples, with default parameters, and the GRCh38 reference file provided. Then, Velocyto (25) was run using the run10X-command, providing as input, in addition to the Cell Ranger output, the genome annotation file from the Cell Ranger GRCh38 reference genome. In addition, as advised by the Velocyto tutorial, the repeat regions were masked and a repeat masker file was downloaded from UCSC genome browser, and provided as input.

To benchmark the effectiveness of QClus against six previously published methods of nuclei filtering, in addition to an adapted implementation of a decontamination method, each method was run for all samples through a custom pipeline, which outputted the list of barcodes to keep. All methods, including QClus, were executed with their default parameters whenever possible, following available tutorials in their respective documentation. Specific input was made only when required or to ensure consistency across comparisons. Details about specific parameter settings for each method are given below. As a base filter, Cell Ranger (11) default filtering (whenever a method did not specifically call for the unfiltered count matrix as input), in addition to conservative thresholds of commonly used quality control (QC) metrics (total counts, total genes and ‘mitochondrial percentage’), was applied. This ensures applicability of the benchmark results. Exact values for these thresholds are given in Supplementary Table S1.

The brain dataset was obtained from GSE243639 (26). Preprocessing was performed the same way as for the heart datasets.

The dataset was then processed using QClus default parameters with the following exceptions. First, ‘minimum_genes’ was set to 400 (based on the dataset distribution of that parameter). Second, since CMs are not present in the brain, the ‘cytoplasm-enriched CM-specific gene expression fraction’ and ‘nuclear-enriched CM-specific gene expression fraction’ metrics were excluded. Third, the cell-type-specific marker genes used to calculate the non-CM specific metric were updated to reflect the different tissue type. We used gene sets provided by the HuBMAP (27) Azimuth web application (https://azimuth.hubmapconsortium.org/references/). Fourth, since there is no CM cluster to identify, the *k* parameter for *k*-means was set to three instead of four. Fifth, since there is no CM cluster, the reference cluster for the outlier filter was selected to be the cluster with the highest mean 'unspliced fraction' value. All other settings were set to default values.

### QClus

#### Initial filter

To remove droplets with poor QC metrics, the lower bound of the number of detected genes is set at 500, the upper bound of the number of detected genes at 6000 and the initial cutoff for mitochondrial percentage to be 40%. The initial number of detected genes parameter can be adjusted based on the overall count distribution of the dataset that has to be filtered.

#### Clustering features: unspliced fraction

After annotating reads as ‘spliced’, ‘ambiguous’ or ‘unspliced’ using Velocyto (25), a ‘fraction_unspliced’ metric is calculated for each droplet. It is the total number of unspliced reads over the total number of reads (spliced, unspliced and ambiguous) in each droplet.

We have also implemented our own method to calculate this metric from the Cell Ranger (11) output, thus removing the need to install and run Velocyto to run QClus. It is included in our Python package and comes with its installation.

#### Clustering features: *mitochondrial percentage*

This metric corresponds to the percentage of reads aligning to the mitochondrial genome (*MT-ND1, MT-ND2, MT-CO1, MT-CO2, MT-ATP8, MT-ATP6, MT-CO3, MT-ND3, MT-ND4L, MT-ND4, MT-ND5, MT-ND6* and *MT-CYB*), for each droplet. The metric is calculated using the Scanpy (28) function calculate_qc_metrics on raw counts.

#### Clustering features: *nuclear fraction*

This metric corresponds to the fraction of reads aligning to the following genes: *MALAT1, NEAT1, FTX, FOXP1, RBMS3, ZBTB20, LRMDA, PBX1, ITPR2, AUTS2, TTC28, BNC2, EXOC4, RORA, PRKG1, ARID1B, PARD3B, GPHN, N4BP2L2, PKHD1L1, EXOC6B, FBXL7, MED13L, TBC1D5, IMMP2L, SYNE1, RERE, MBD5, EXT1* and *WWOX*. These were chosen as they exhibit high correlation with *MALAT1*, which is known to be highly expressed in the nucleus. The metric is calculated using the Scanpy function calculate_qc_metrics () on logarithmized counts. Logarithmization is performed using the Scanpy function log1p ().

#### Clustering features: nuclear CM marker genes

A set of genes found to be specifically expressed in CMs, but absent from ambient RNA contamination were selected as highly expressed in CM nuclei: *RBM20, TECRL, MLIP, CHRM2, TRDN, PALLD, SGCD, CMYA5, MYOM2, TBX5, ESRRG, LINC02248, KCNJ3, TACC2, CORIN, DPY19L2, WNK2, MITF, OBSCN, FHOD3, MYLK3, DAPK2* and *NEXN*. Droplets with a high level of expression of these genes are expected to contain CM nuclei. The metric is calculated using the Scanpy function calculate_qc_metrics () on raw counts. These genes were derived from cell-type marker discovery from https://www.biorxiv.org/content/10.1101/2021.06.23.449672v2.abstract (see supplementary figures for violin plots showing the genes in question and their specificity).

#### Clustering features: cytoplasmic CM marker genes

A set of genes found to be specifically expressed in CMs, but present in high level from ambient RNA contamination were selected as highly expressed in CM cytoplasm: *TTN, RYR2, PAM, TNNT2, RABGAP1L, PDLIM5, MYL7* and *MYH6*. The metric is calculated using the Scanpy function calculate_qc_metrics () on raw counts. These genes were derived from cell-type marker discovery from https://www.biorxiv.org/content/10.1101/2021.06.23.449672v2.abstract (see supplementary figures for violin plots showing the genes in question and their specificity).

#### Clustering features: cell-type-specific fractions

For each of the 11 remaining cell types, a set of genes was selected as markers of these cell types from Wilcoxon rank sum test differential expression analysis (Supplementary Table S1). The fraction of reads aligning to those sets of genes was calculated for each droplet, using the Scanpy function calculate_qc_metrics () on raw counts. Next, for each droplet, the maximum value out of those 11 metrics was selected. These genes were derived from cell-type marker discovery from https://www.biorxiv.org/content/10.1101/2021.06.23.449672v2.abstract.

#### Clustering method

Once the clustering features have been computed for the remaining nuclei, the features are scaled using the MinMaxScaler function implemented in the scikit-learn (29) package. Next, the *k*-means algorithm was used, as implemented in the scikit-learn package, to partition the nuclei into four clusters, using all six clustering features. By default, the cluster demonstrating the lowest mean value of ‘unspliced fraction’ is removed, as this cluster is predicted to contain empty droplets. However, the user can choose to remove any number of clusters. The default number of clusters is four, but that number can be changed to adapt the software to different contamination profiles (Supplementary Figure S1). The list of clustering features can also be modified, to allow for adaptation of QClus for other tissue types than heart (as exemplified in the brain dataset that we process).

#### Outlier filtering

Filtering threshold values are calculated for the fraction of ‘unspliced reads’ in addition to mitochondrial percentage, based on the distribution of these metrics in the r non-CM cluster, defined as the cluster with the highest mean value for the pct_counts_nonCM metric. The unspliced fraction threshold is chosen as the lower quartile minus 0.1. The mitochondrial percentage threshold is chosen as the upper quartile plus 5%. These values can be adjusted by the user. Droplets are then filtered out if they go beyond these thresholds in both metrics (below the threshold for unspliced fraction and above for mitochondrial percentage).

#### Doublet removal

The Scrublet algorithm is used in each sample, and droplets whose doublet score exceeds 0.1 are removed. This filters out both doublets and highly contaminated droplets, whose distribution can resemble doublets (Supplementary Figure S2).

### Other methods

#### Traditional threshold filtering based on QC metrics only

As a baseline, we employed standard QC filtering (threshold filtering) that is routinely run in snRNA analysis, and often not accompanied by more filtering methods. Metrics used were mitochondrial percentage, total counts and total genes. The exact threshold values utilized were set based on the details given in the respective studies and can be reviewed in Supplementary Table S2.

#### DIEM

DIEM (6) is a droplet filtering method that is based on a model of RNA expression, taking into account contamination and cell-type-specific patterns, using a multinomial distribution. The initialization phase requires users to set a stringent count threshold based on a barcode rank plot, subsequently tagging nuclei below this threshold as debris, meaning they are expected to represent the profile of ambient RNA contamination. Using the expectation-maximization algorithm, DIEM determines model parameters. Each droplet receives a score reflecting its expression of genes attributed to the debris set, enabling users to implement further filtration based on the debris score. The debris score was calculated using default settings and a debris score cut-off of 0.5, which is the default value, was set to filter out empty and highly contaminated droplets.

#### Modified DecontX

DecontX (21) employs a Bayesian framework to decipher the distribution of background contamination and ascertain contamination levels in each droplet. This model is then used to remove contamination from the gene expression matrix. In this study, we focus on droplet filtering and not decontamination *per se*. Thus, the contamination score derived from DecontX results was used to filter droplets. Droplets that had a contamination score of more than 0.43 were removed. The threshold was established to compare DecontX results with QClus results, as it resulted in the same number of droplets being filtered out in the PERIHEART (30) dataset in both methods.

#### EmptyNN

EmptyNN (17) is a cell-calling algorithm to differentiate between droplets that contain cells and those that are empty or cell-free. It utilizes a PU (Positive-Unlabeled) learning bagging strategy, based on the idea that barcodes with very low total counts are likely to represent genuine cell-free droplets.

#### SampleQC

SampleQC (18) fits a Gaussian mixture model spanning multiple samples, subsequently filtering outlier nuclei. The metrics we used as inputs were counts, genes, mitochondrial percentage and unspliced fraction. The ‘*k*_all’ argument was set to ‘*k*_all = 2’, which was observed to fit the data well in most cases. The method suggests testing different values of that parameter between groups of samples. However, further optimization is outside of the scope of our study, as it renders benchmarking more subjective: we thus concentrate on fully unsupervised methods, and setting parameters to their default value.

#### DropletQC

DropletQC (19) utilizes both splicing fraction and total detected UMIs to discern among empty droplets, intact nuclei and contaminated nuclei. Estimated thresholds are employed to identify nuclei positioned above or below these designated values. We executed the identify_empty_drops function of the package to identify and remove empty droplets.

#### CellBender

CellBender (31) is a tool designed to decontaminate data by constructing a probabilistic model to differentiate between true biological counts and background noise in the observed feature count matrix, using a deep generative model. CellBender was run with the expected-cells parameter set to 5000, the total-droplets-included parameter set to 40 000, the *fpr* parameter set to 0.01 and the epochs parameter set to 150. CellBender uses its modeling of contamination to identify empty and non-empty droplets, in addition to the decontamination itself. This droplet selection feature was used for the benchmarking. However, the contamination removal part of the output was not used, and the original count matrix is used to calculate quality metrics for benchmarking.

### Benchmarking

After running each of the droplet filtering methods on all samples, for all datasets, quality metrics were calculated at the sample-level. Standard scaling was performed with StandardScaler from scikit-learn to standardize quality metrics for each unique sample. Each piece of sample data was isolated and scaled independently. Embeddings for each sample–method combination were constructed using Scanpy, using standard dimension reduction and embedding procedures, as laid out in the Scanpy tutorial. The sc.pp.filter_genes function filtered genes in the AnnData object present in <10 nuclei. Subsequently, highly variable genes were identified using the sc.pp.highly_variable_genes function with parameters min_mean = 0.005, max_mean = 5 and min_disp = 0.5. The dataset was then narrowed down to these highly variable genes. Data corrections for total counts and the percentage of mitochondrial counts were applied with sc.pp.regress_out. The sc.pp.scale function scaled the data to a maximum value of 10.

## Results

### Description of datasets

To understand how contamination is distributed in snRNA-seq data, we set out to explore the distribution of the known contamination metrics, such as mitochondrial percentage, as well as to develop novel metrics specific to the tissue type of interest (e.g. human heart tissue) that could be used during the quality control step of the workflow.

To explore the distribution of contamination metrics in human heart snRNA-seq datasets and benchmark droplet filtering methods, we collected six datasets. They consisted of four external datasets and two datasets of our own. The Chaffin *et al.* (24) data comprise 95 samples from the left ventricles of 11 patients with dilated cardiomyopathy, 15 with hypertrophic cardiomyopathy and 16 control hearts. The Hill *et al.* (22) data provide 30 samples from nine pediatric patients with congenital heart disease and four control hearts. The Koenig *et al.* (23) data include 35 samples from the left ventricles of 17 patients with dilated cardiomyopathy and 28 control hearts. The Litviňuková *et al.* (5) data have 42 samples from six anatomical cardiac regions from seven healthy hearts. Our own two datasets originate from our previous study, Linna-Kuosmanen *et al.* (30), including 50 right atrial appendage samples, from 15 patients with ischemic heart disease, nine with myocardial infarction, 11 with ischemic heart failure, three with non-ischemic heart failure and 10 control patients. These samples originate from the CAREBANK and PERIHEART datasets.

### Description of contamination metrics

Two well-established universal snRNA-seq contamination metrics are the unspliced and mitochondrial percentage (Figure 1B). As the names suggest, the unspliced fraction measures the fraction of unspliced reads observed in the droplet and the mitochondrial percentage measures the fraction of reads originating from mitochondrial genes. Given the higher number of unspliced transcripts in the nucleus compared with cytoplasm, the metric tied to it is anticipated to show an inverse correlation with contamination, whereas mitochondrial pecentage is expected to positively correlate with contamination, as mitochondria are only present in the cell cytoplasm. Both metrics have been established and utilized in previous research to measure ambient RNA levels (6,15,18,19). Expectedly, our findings in the heart data confirmed a strong negative correlation between the two metrics (Figure 1C), and the Uniform Manifold Approximation and Projection (UMAP) plots of the samples showed a central cluster exhibiting high levels of mitochondrial and low levels of unspliced fraction (Supplementary Figure S3), corresponding to putative empty and highly contaminated droplets, thereby confirming the value of these metrics for measuring ambient RNA levels.

The third metric used in our study was the ‘nuclear fraction’ (Figure 1B), which represents a nuclear-enriched gene expression fraction (6,19). *MALAT1* produces a transcript that localizes to nuclear speckles (32) and is believed to regulate the distribution and activity of splicing factors within these speckles. As nuclear-enriched genes possess a relative expression that should decrease in droplets with more cytoplasmic RNA, this fraction is expected to be inversely correlated with ambient RNA contamination in snRNA-seq. Consistent with previous literature (20), we observed positive correlation with unspliced fraction and negative correlation with mitochondrial percentage for the metric (Figure 1C).

### CMs as a source of contamination

CMs, one of the most abundant cell types in cardiac tissue, possess a high RNA content in their cytoplasm due to their size and function. Accordingly, we observed CM-expressed genes to account for an important amount of the ambient RNA contamination in the samples (Supplementary Figures S4 and S5). Thus, we hypothesized that some of these genes could be used as contamination metrics, provided that the method would not falsely filter out real CM nuclei.

To take this into account, we first needed to define a metric that would help us to distinguish CMs from non-CMs. Droplets that contain a single non-CM nucleus will have high levels of gene expression corresponding to marker genes from the non-CM cell type. We thus defined a metric called ‘non-CM-specific gene expression fraction’ (Figure 1B), representing the fraction of reads aligning to the most highly expressed non-CM marker gene set (details, including the definitions of gene sets, described in methods). Furthermore, we observed that genes that were specific to other cell types than CMs contributed to contamination at a much lower level than CMs (Supplementary Figure S5). A droplet that was enriched in one of the gene sets constituted by cell-type-specific marker genes was very likely to contain a nucleus of that cell type, instead of being empty. It was also less likely to be highly contaminated, since that would drive down this percentage. We confirmed this by the correlations with unspliced fraction and mitochondrial percentage in Figure 1C.

Droplets that contain a single CM nucleus are expected to display a high level of CM-specific gene expression. However, it can be difficult to distinguish true, high-quality CMs from contaminated droplets (either empty or containing a CM or non-CM nucleus) due to the high level of expression of CM-specific genes in the contamination. Interestingly, we observed CM-specific genes diverging into two groups: those contributing heavily to contamination and those contributing less (Supplementary Figure S6), putatively corresponding to cytoplasm- and nuclear-enriched genes, respectively. Derived from this observation, we then defined two additional metrics: ‘cytoplasm-enriched CM-specific gene expression fraction’ and ‘nuclear-enriched CM-specific gene expression fraction’ (Figure 1B).Cytoplasm-enriched CM-specific gene expression fraction can serve as a metric of contamination, as demonstrated by its strong correlation with mitochondrial percentage and negative correlation with unspliced fraction (Figure 1C). Conversely, nuclear-enriched CM-specific gene expression fraction helped to distinguish true CM-containing droplets with low contamination from empty or highly contaminated droplets that may still contain significant amounts of CM marker gene expression due to the high contribution of CM-specific genes to ambient background RNA (Figure 1C).

### Contamination metric values vary within and between datasets before preprocessing

After exploring and creating contamination metrics based on their specific distribution across droplets, we investigated their global distribution patterns across samples and datasets (Figure 1D) to establish an automated, universal filtering method. We observed a wide range of values for the created metrics within and across datasets, demonstrating the need for a method that automatically applies a flexible approach dependent on the sample-specific expression patterns and contamination levels. Full results for the mean values of the above quality metrics in unfiltered samples across the 252 samples can be found in Supplementary Table S3.

### QClus efficiently removes empty droplets and highly contaminated nuclei

**Figure 2.**
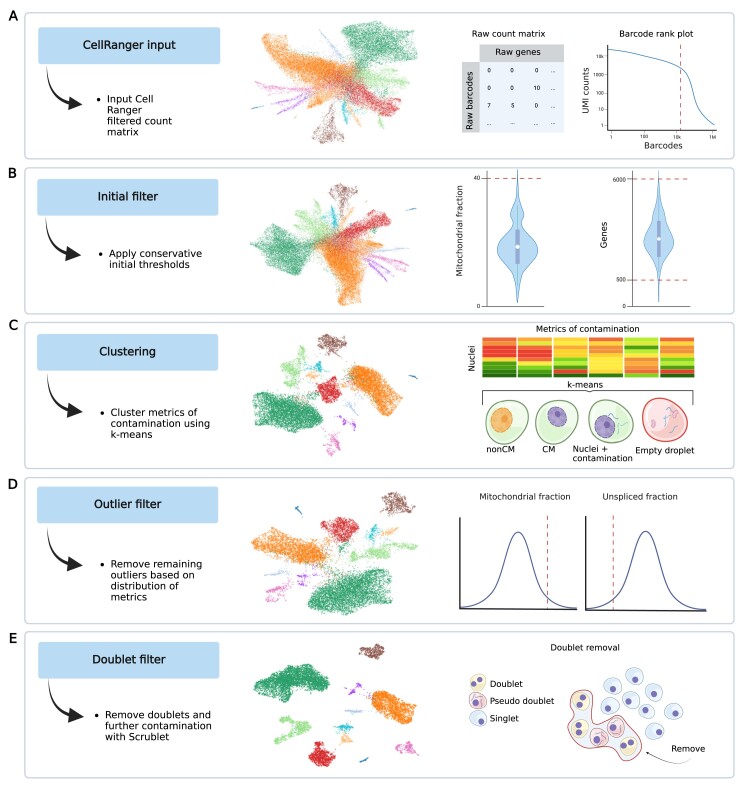
Overview of empty and highly contaminated droplet removal using QClus. The UMAP progression shows sample CB-S00 from the CAREBANK dataset. (**A**) The QClus algorithm begins with the pre-filtered count matrix from Cell Ranger (11). (**B**) A conservative initial filter is applied to remove tail ends of known quality metrics. (**C**) General as well as cell-type-specific quality metrices are used for *k*-means clustering of droplets. The cluster displaying the lowest quality is removed by default. The user can choose to remove further clusters. (**D**) An outlier filter based on the underlying distribution of mitochondrial percentage and unspliced fraction. (**E**) Doublet filtering is applied to remove doublets as well as droplets exhibiting doublet-like expression due to the presence of a non-CM nucleus and CM-derived ambient RNA. Created in BioRender [Linna-kuosmanen, S. (2023); https://BioRender.com/f33a459].

To answer the need for an automated method that can handle sample-specific expression patterns and contamination levels, we built QClus. QClus is centered on clustering droplets based on their defined quality metrics (Figure 1C). In addition, it uses CM-specific and non-CM-specific gene expression patterns as well as nuclear and cytoplasmic gene expression fractions in the metrics to distinguish CM nuclei, non-CM nuclei, highly contaminated droplets and empty droplets, further improving the clustering. The QClus pipeline is divided into five steps:

**Step 1**: Input data. The pipeline starts with data filtered by Cell Ranger (10X Genomics) (11), which attempts to remove empty droplets using an algorithm based on the EmptyDrops (10) method (Figure 2A). The Cell Ranger algorithm identifies low-RNA content cells in samples with mixed cell populations. It initially uses a UMI count-based cutoff to detect high-RNA content cells, followed by a detailed RNA profile analysis of remaining barcodes to separate actual cells from empty droplets. To illustrate this, we used a sample from the CAREBANK (30) dataset as an example. After Cell Ranger filtering, the nuclei were found in a star shape on the dimension reduction map (UMAP), where all cell types seemed to originate from a common cluster. We hypothesized that the star center was composed of empty droplets and highly contaminated nuclei, as the cell types in the adult human heart are differentiated and therefore expected to form independent clusters. This hypothesis is confirmed by the high level of mitochondrial percentage and low level of unspliced fraction in the star center (Supplementary Figure S3).

**Step 2**: Initial filtering. The second filtering step removes clearly empty and low-quality droplets based on the detected number of genes and mitochondrial percentage, using high thresholds. The gene-level filtering ensures that all nuclei have enough genes to be of significant biological interest in the downstream analyses, whereas a high mitochondrial threshold is used to remove clear outliers (Figure 2B).

**Step 3**: *K*-means clustering. In this step, the method calculates the six contamination metrics defined earlier (Figure 1B; details in ‘Materials and methods’ section). These values are then used as input for *k*-means clustering to identify four clusters (Figure 2C; Supplementary Figure S7): (i) non-CM nuclei with low levels of contamination, low CM-specific gene expression, both nuclear and cytoplasmic and high non-CM-specific gene expression (Supplementary Figure S8); (ii) CM nuclei with low levels of contamination, high CM-specific gene expression, both nuclear and cytoplasmic and low non-CM-specific gene expression (Supplementary Figure S9); and (iii) highly contaminated nuclei (Supplementary Figure S10) and (iv) empty droplets (Supplementary Figure S11), both of which have high contamination, high cytoplasmic CM-specific gene expression, low nuclear CM-specific gene expression and low non-CM-specific gene expression. The default settings instruct QClus then to remove the empty droplets cluster only, but the user can choose to also remove the highly contaminated nuclei cluster, on a sample-by-sample basis (Supplementary Figure S1). In most samples this step will remove the highest number of droplets and show the most significant improvements in sample quality.

**Step 4:** Outlier filtering. The fourth filtering step removes highly contaminated nuclei in a more adjustable manner by identifying additional outliers based on the unspliced fraction and mitochondrial percentage distribution within the sample (Figure 2D). In this process, outliers are selected and filtered based on a threshold determined by quantiles of the distribution within the non-CM cluster, defined as the cluster showing the highest level of the non-CM marker gene expression metric. This stage identifies and excludes those nuclei that deviate substantially from the expected distribution of the two metrics. It allows for a finer control over the quality and number of retained nuclei. Our method proposes default parameters for this step, used throughout the paper, which are meant to provide balanced results.

**Step 5:** Doublet filter. To finalize the filtering, our pipeline uses the Scrublet (33) algorithm, which removes doublets. However, in addition to removing doublets, the Scrublet algorithm also removes highly contaminated nuclei in the heart, as remaining highly contaminated nuclei can appear as doublets (Figure 2E). These pseudo-doublets are non-CMs that have high levels of contamination (Supplementary Figure S12) and also appear to contain CM-derived RNA (Supplementary Figure S2).

After QClus preprocessing, the final UMAP showed a clear separation of the 11 major cell types observed in the illustrated sample. No single metric alone predicted which nuclei were removed and which were kept (Supplementary Figure S13), confirming that our multi-metric approach taken during the quality control maximizes the biological signal for downstream analyses.

### QClus outperforms other methods across multiple distinct quality metrics

To test our hypothesis regarding the effectiveness and versatility of the QClus method, we performed a comparative analysis of its performance against seven alternative droplet filtering methods across the six heart snRNA-seq datasets. QClus was run without the doublet filtering step to provide a fair comparison against other methods which do not include doublet filtering. In addition, QClus was set to its default parameters regarding Step 3, removing only the empty droplet cluster in this step (Supplementary Figure S8), allowing for fully automated execution. Utilizing the six datasets, we selected four distinct quality metrics for evaluation: unspliced fraction, mitochondrial percentage, total counts and the number of genes expressed (for full results, see Supplementary Table S4). For each sample, the metrics were standardized across methods (for full results, see Supplementary Table S5). This normalization ensures the standardized comparison of the performance of each method, across all samples.

**Figure 3.**
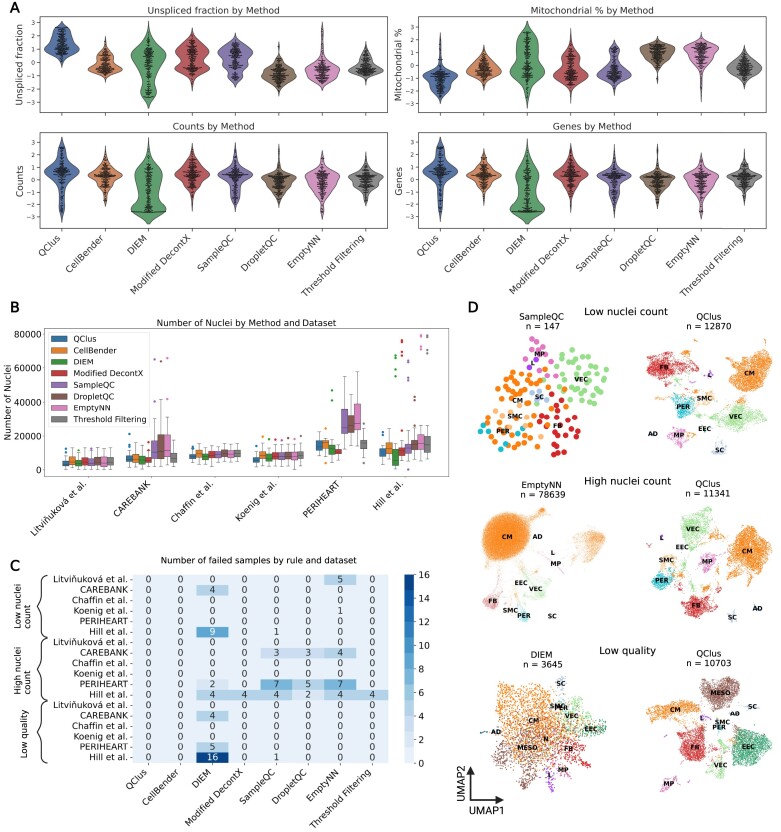
Benchmarking QClus against alternative methods shows improved quality metrics and robust nuclei retention. (**A**) Comparison of achieved quality for six published methods and a control method with traditional quality metric threshold filtering (each sample is a datapoint). (**B**) Number of retained nuclei by method. (**C**) Failed samples by rule and dataset. (**D**) Three examples of failed samples by alternative methods compared against the same samples processed with QClus. The three samples are: Hill *et al.*, sample Hi_WU198RV_rep1 (top row), Hill *et al.*, sample Hi_WU13235_rep1 (middle row) and CAREBANK sample CB-Q34 (bottom row).

Across all four QC metrics, QClus displayed the highest number of samples with the best result for that metric (Figure 3A), outperforming other methods in 138 out of 252 samples (54.76%) for unspliced fraction, in 116 samples (46.03%) for mitochondrial percentage, 78 samples (30.95%) for total counts and 96 samples (38.10%) for the mean number of genes expressed per nuclei.

Another important metric in the assessment of droplet filtering methods is the resulting number of kept nuclei. Due to the distribution of quality metrics across nuclei, a more stringent threshold decreases the number of nuclei and increases their quality. There is thus an inherent trade-off between the number of nuclei retained and the quality achieved. While retaining more nuclei can potentially enhance the richness of the dataset, it might do so at the expense of quality, which could lead to noise or false conclusions. Conversely, being overly stringent might result in the omission of high-quality nuclei, thereby limiting the scope of insights that could be drawn. Investigation of this aspect across the datasets and methods revealed that QClus, although retaining fewer nuclei compared with some methods, such as EmptyNN (17), DropletQC (19) and SampleQC (18), remains within the expected range for nuclei retention across various datasets (Figure 3B). In addition, compared with other methods, the nuclei retention counts appeared more stable. Given the consistent nature of experimental procedures within each dataset, large fluctuations of nuclei retention could be indicative of less reliable filtering.

To further benchmark and compare the eight preprocessing methods, we established criteria that, when fulfilled, would indicate a processing failure—i.e. an unacceptable result for the respective sample. The method-wise numbers on how many samples failed for each rule are shown in Figure 3C and Supplementary Table S6. The first criteria we considered was ‘low nuclei count’, which flagged cases where a considerably greater number of nuclei of comparable quality could have been retained, i.e. any method that retained <3000 nuclei for a particular sample, while another method was able to retain over four times as many nuclei with an unspliced fraction within 10% for the same sample. Twenty sample–method cases fell into this category. These failures were spread across DIEM (6) (13 samples), EmptyNN (17) (six samples) and SampleQC (18) (one sample). An example from the Hill *et al.* (22) dataset is shown in Figure 3D, where SampleQC retained 147 nuclei, while QClus was able to retain 12 870 good-quality nuclei (another example can be seen in Supplementary Figure S14).

The second criterion we considered was ‘high nuclei count’, which flagged samples that retained more than 30 000 nuclei after preprocessing. Given the experimental design of the datasets, parameters of the preprocessing methods [such as the ‘expected cells’ parameter of CellBender (31)] used in the publications, and observed ranges of retained nuclei in the original publications, samples were expected to contain between 3000 and 10 000 nuclei. Of the analyzed sample–method combinations, 53 met this condition, signaling a failure in quality control. These failures were distributed across six methods: Modified DecontX (21) (four samples), DIEM (6) (six samples), DropletQC (19) (10 samples), EmptyNN (17) (15 samples), traditional preprocessing (four samples) and SampleQC (18) (14 samples). For example, in a Hill *et al.* (22) sample (Figure 3D), EmptyNN retained 78 639 nuclei, while QClus retained 11 341 nuclei (another example shown in Supplementary Figure S14).

The third criterion we used was ‘low quality’. A method was considered to have failed, when the median unspliced fraction for the retained nuclei of a method–sample combination was <0.3, and another method retained a greater number of nuclei from the same sample with a median unspliced fraction of at least 0.2 higher. Thus, the same number of nuclei of significantly higher quality could be identified using an alternative method. Twenty-six sample–method combinations failed using this criteria, namely DIEM (6) (25 samples) and SampleQC (18) (one sample). For example, in a CAREBANK sample (Figure 3D), DIEM retained 3645 nuclei with a mean unspliced fraction of 0.22 and no clear cell-type separation on the UMAP, while QClus retained 10 703 nuclei with an unspliced fraction of 0.64 and clear separation (another example shown in Supplementary Figure S14).

**Figure 4.**
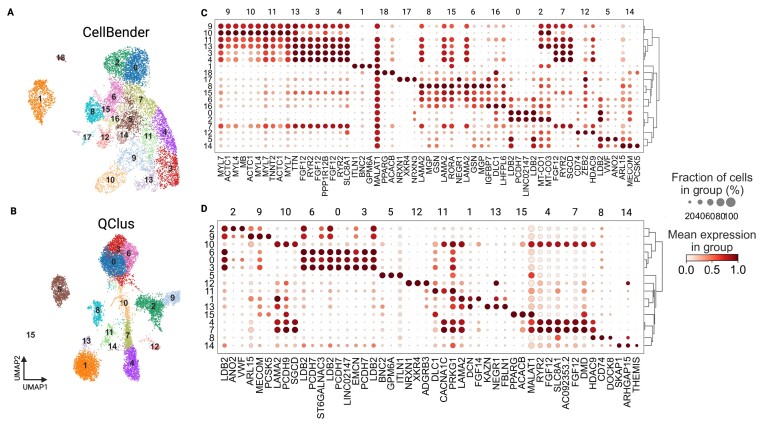
Comparison of CellBender and QClus processing. The sample is from the PERIHEART dataset, sample PH-V63. (**A** and **C**) UMAP and dotplot for CellBender processing of this sample. (**B** and **D**) UMAP and dotplot of QClus processing. Dot plots show the top three genes per Leiden group.

Taken together, QClus outperformed other methods by displaying the highest number of samples with the best results in quality while remaining within the expected range for nuclei retention across various datasets, with more stable nuclear retention counts compared with other methods. Based on the criteria for processing failures, only two of the methods, QClus and CellBender (31), exhibited no failures across any of the six datasets. However, while CellBender processed samples passed the set failure criteria, there were samples in which it did not perform well. These same samples were found to be processed better with QClus. In an example shown in Figure 4, CellBender resulted in low cell-type separation and expression signal (Figure 4A and C), whereas after QClus, cell types were found in higher abundance and balanced composition (Figure 4B and D).

### The versatility and effectiveness of QClus is confirmed in a brain dataset

To illustrate that QClus performs in non-cardiac tissues as well, we acquired a publicly available brain snRNA-seq dataset, consisting of 28 samples. This dataset is composed of ‘post-mortem’ tissue from 15 individuals with sporadic Parkinson’s disease and 14 control subjects. Due to the lack of CMs in brain tissue, the two CM-specific metrics (cytoplasm-enriched CM-specific gene expression fraction and nuclear-enriched CM-specific gene expression fraction) were omitted, and cell type scores were calculated using adjusted gene lists (details in the ‘Materials and methods’ section).

**Figure 5.**
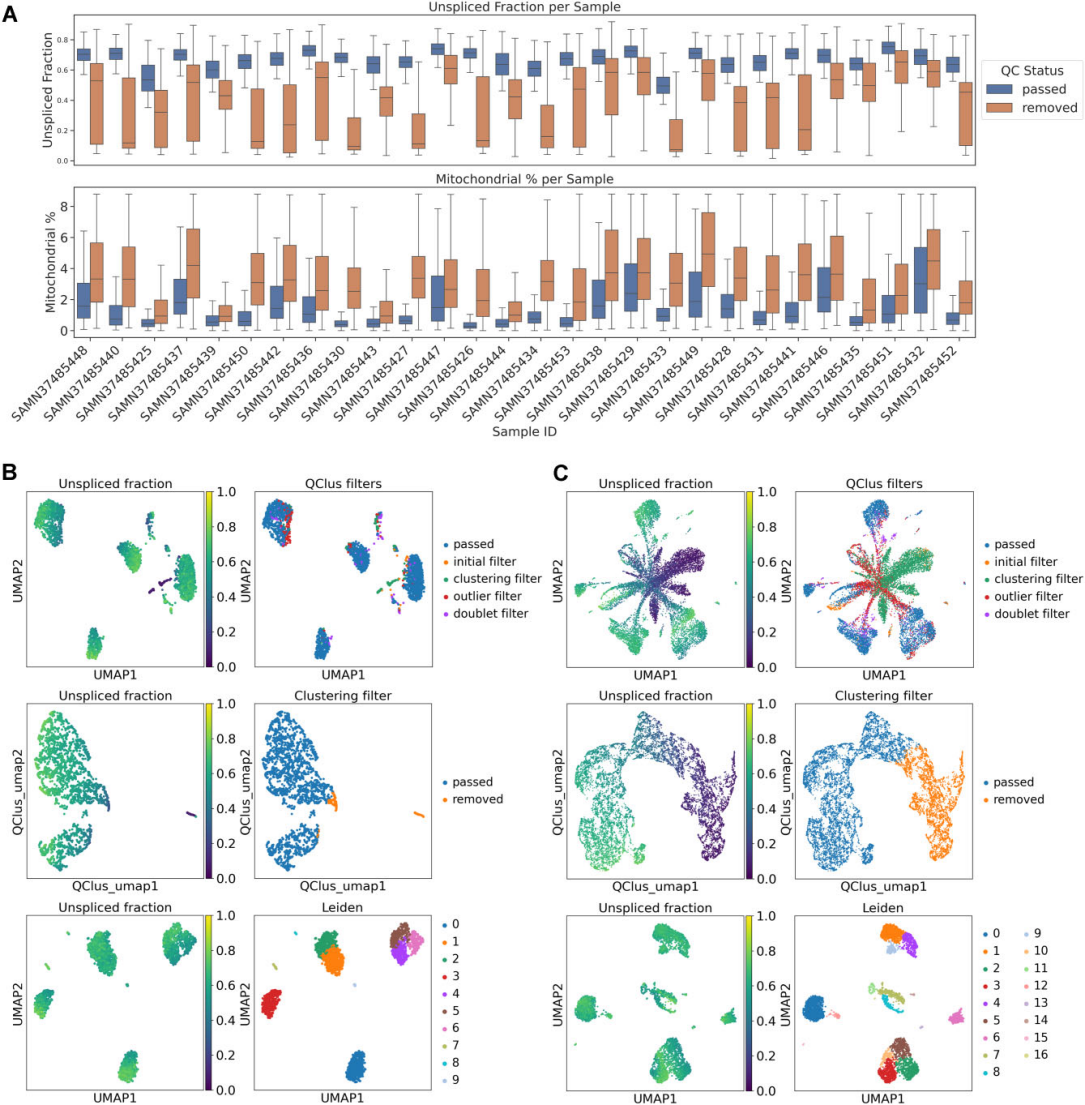
Evaluation of QClus performance in a brain dataset. (**A**) Box plots of contamination metrics by filter condition across all samples in the dataset. Mitochondrial percentage is truncated at the 90th percentile to remove the highest 10% of values for each metric for visualization purposes. (**B** and **C**) Example of QClus processing of two samples (left panel: sample SAMN37485439, right panel: sample SAMN37485434) exhibiting high quality (B) and low quality (C) prior to filtering. The first row shows a UMAP of the Cell Ranger -filtered count matrix. The left column shows the distribution of unspliced fraction and the right column shows which droplets QClus flagged for removal during processing. The second row shows UMAPs for the six clustering metrics. After all metrics are calculated for all droplets, the UMAP dimensions are calculated to visualize the clustering feature space. Left is the distribution of unspliced fraction and right is which droplets the QClus clustering filter flagged for removal. The third row shows the resulting UMAP of the count matrix after QClus-flagged droplets were removed.

Similar to heart samples, QClus successfully retained high-quality nuclei while removing lower quality droplets (Figure 5A). Across all samples, retained droplets showed high unspliced fraction and low mitochondrial percentage compared with filtered droplets, although a significant variation in sample quality was observed in the dataset prior to filtering (Figure 5B and C). The results showed that in low pre-filtering contamination, very few droplets were removed (Figure 5B), whereas in high contamination (Figure 5C), the star-shape pattern familiar from the cardiac samples was seen, leading to large number of low-quality droplets being removed. In both cases, the resulting UMAPs showed a good cluster separation in the final stage. Since all samples were run automatically with default parameters settings, this further demonstrates the ability of QClus to handle significant levels of variation in terms of quality without extensive supervision. Results for the brain dataset, with averaged values for all quality metrics, can be found in Supplementary Table S7.

As with cardiac samples, we found that many samples in the brain dataset exhibited a very low-quality center of the star-shape. When plotting the space of contamination metrics on a UMAP, this would often be visible as a large cluster of near-zero unspliced fraction droplets (Supplementary Figure S15).

## Discussion

QClus is a droplet filtering method that is tailored to overcome unique challenges in snRNA-seq processing tissue-specifically. The benchmarking results advocate for specialized preprocessing, especially for samples that show high levels of background contamination.

The strength of QClus lies in its ability to process heterogeneous datasets in an automated way while adapting to variations in sample characteristics, such as nuclei number, overall quality and contamination levels. The adaptability reduces the need for meticulous manual oversight required in other similar methods, and our benchmarking attests to the performance of QClus in unsupervised scenarios. The flexibility of the method allows the parameters of the algorithm to be tweaked globally or at the level of individual samples, providing researchers with the opportunity to balance between nuclei retention and contamination control, based on the specific biological questions at hand.

As the method is designed to work tissue-specifically, we focused here on one specific tissue type known to be challenging, the human heart, and geared our approach toward heart-specific features that our research corroborated. However, the method is adaptable to other tissue types by modifying the list of marker genes or the input metrics, as demonstrated by its performance in the brain dataset. This is especially important, as recent results from Montserrat-Ayuso and Esteve-Codina (16) suggest that several published cell atlases included large clusters of droplets that are likely to be empty, but were not successfully removed during quality control. This leads to them being incorrectly annotated as cell types, making further findings based on this data less reliable and highlighting the need for a method that successfully removes these droplets. In addition to Montserrat-Ayuso and Esteve-Codina (16), Clarke *et al.* (20) have advocated for the importance of MALAT1 expression threshold for snRNA-seq QC. These studies provide further evidence in favor of our approach, which uses these two metrics in addition to mitochondrial percentage as part of its QC features.

As QClus is specifically designed to function without supervision and tailored for high-throughput datasets, where manual intervention is not feasible, it is plausible that some benchmarked methods would have shown enhanced performance had they been given more manual adjustments tailored to each sample. However, such a procedure would deviate from our primary objective, which was to identify a method that reliably and automatically works for extensive datasets with minimal manual input. With the advent of novel techniques, the decreasing cost of sequencing, and the generation of larger datasets, there is an unmistakable demand for fully automated methods that can efficiently handle large volumes of data while maintaining accuracy and reliability. However, the parameters of the method are fully customizable, allowing it to be tailored to the needs of the user, if such need arises. This is especially exemplified in the brain dataset, where cell-type-specific features were easily adjusted to fit the tissue type. Thus, our method works well unsupervised, but is still fully adjustable if needed, each step being transparent and interpretable.

QClus, though effective, has room for growth. Accounting for contamination distributed within cell-type populations might improve accuracy, given that we observe varying levels even within non-CMs. Integrating QClus with a decontamination algorithm, such as CellBender (31) or DecontX (21), might further enhance its efficiency, establishing an integrated solution that both filters and adjusts the count matrix. Thus, the optimal strategy could involve a careful and balanced approach to both nuclei calling and decontamination. The specific choice and combination of methods and strategies might be guided by the tissue type, cellular complexity and specific questions asked in a study.

In conclusion, challenging tissues, exemplified by cardiac tissue in our study, highlight the need for tissue-specific preprocessing methodologies that can handle large datasets containing samples of varying qualities in an automated fashion. The idiosyncrasies of contamination across different tissues and even disorders demand tailored solutions. QClus emerges as a potent tool for tissue preprocessing – as shown in cardiac and brain tissues, with promising avenues for adaptation to other tissue types – paving way for more precise, tailored snRNA-seq analyses.

## Supplementary Material

gkae1145_Supplemental_Files

## Data Availability

Datasets for the external dataset, the FASTQ files from each sample were downloaded from their respective repository. The Hill *et al.* (22) and Koenig *et al.* (23) data were obtained from GEO (GSE203275 and GSE183852, respectively). The Litviňuková *et al.* (5) dataset was obtained from ENA (https://www.ebi.ac.uk/ena/browser/view/PRJEB39602). The Chaffin *et al.* (24) data were obtained from dbGaP (https://www.ncbi.nlm.nih.gov/projects/gap/cgi-bin/study.cgi?study_id=phs001539.v1.p1) with proper authorizations. The CAREBANK and PERIHEART data originate from Linna-Kuosmanen *et al.* (https://doi.org/10.5281/zenodo.10822323). The brain dataset was obtained from GSE243639 (26). QClus is a ready-to-use python package, which is suited for integration with Scanpy processed single nuclei data. The current version of code, instructions to download and install the package, as well as tutorials can be found on GitHub (https://github.com/linnalab/qclus). Additionally, the code is also stored on Zenodo (https://zenodo.org/doi/10.5281/zenodo.13773285).
