# Supplementary material for: QClus: a droplet filtering algorithm for enhanced snRNA-seq data quality in challenging samples
Source: Nucleic Acids Res. 2024 Dec 5;53(1):gkae1145. doi: 10.1093/nar/gkae1145 (PMC11724311; doi:10.1093/nar/gkae1145)
Supplement: gkae1145_Supplemental_Files [file gkae1145_supplemental_files.zip › C_QClus supplementary figures.pdf]

SUPPLEMENTARY FIGURES

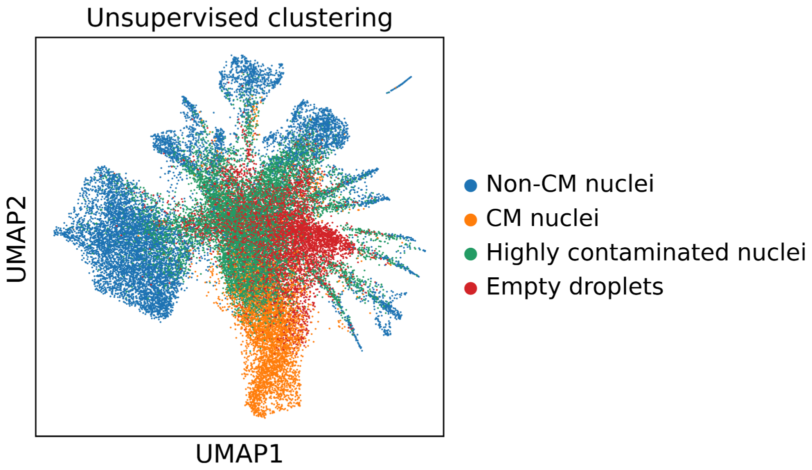

Figure S1. Identified QClus clusters on UMAP of a single sample. The sample is from the CAREBANK dataset, sample CB-S00.

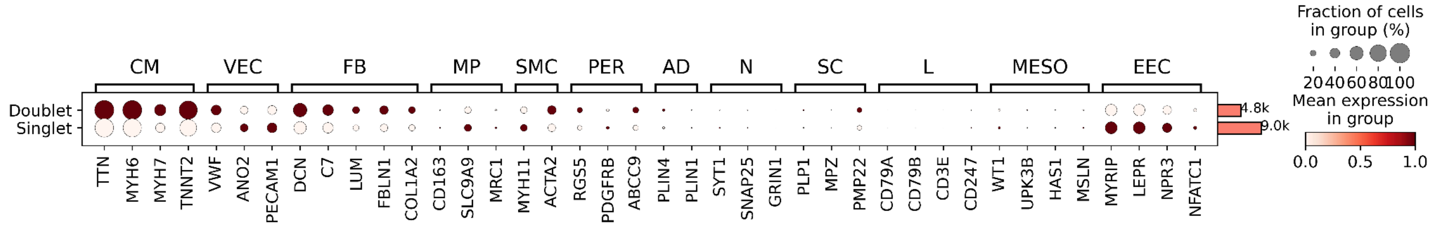

Figure S2. Expression of marker genes in droplets identified as doublets show high expression of CM marker genes. The sample is from the CAREBANK dataset, sample CB-S00.

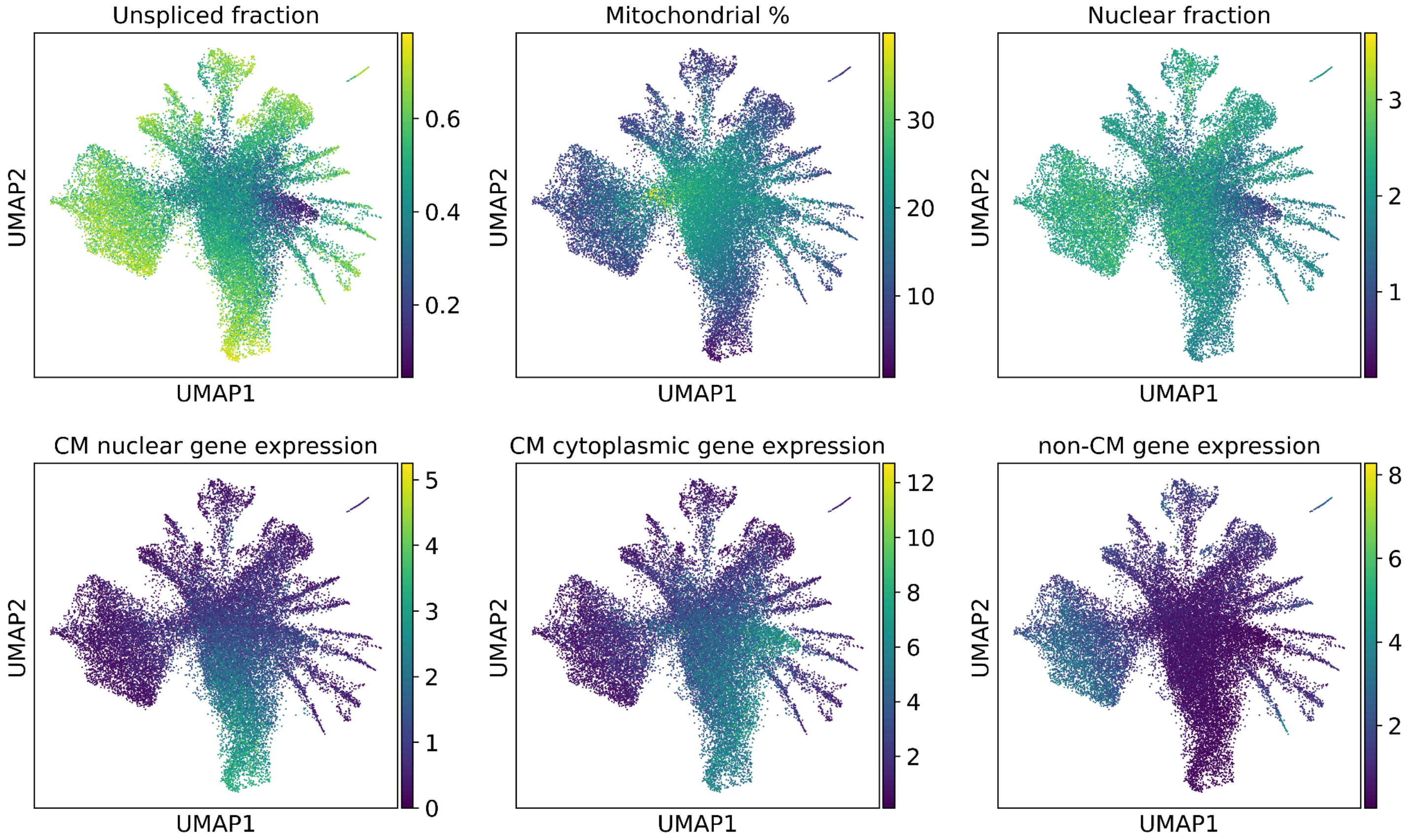

Figure S3. Distribution of the metrics that are used by QClus across the droplets of an unfiltered sample. Shown on a UMAP dimension reduction plot. The sample is from the CAREBANK dataset, sample CB-S00.

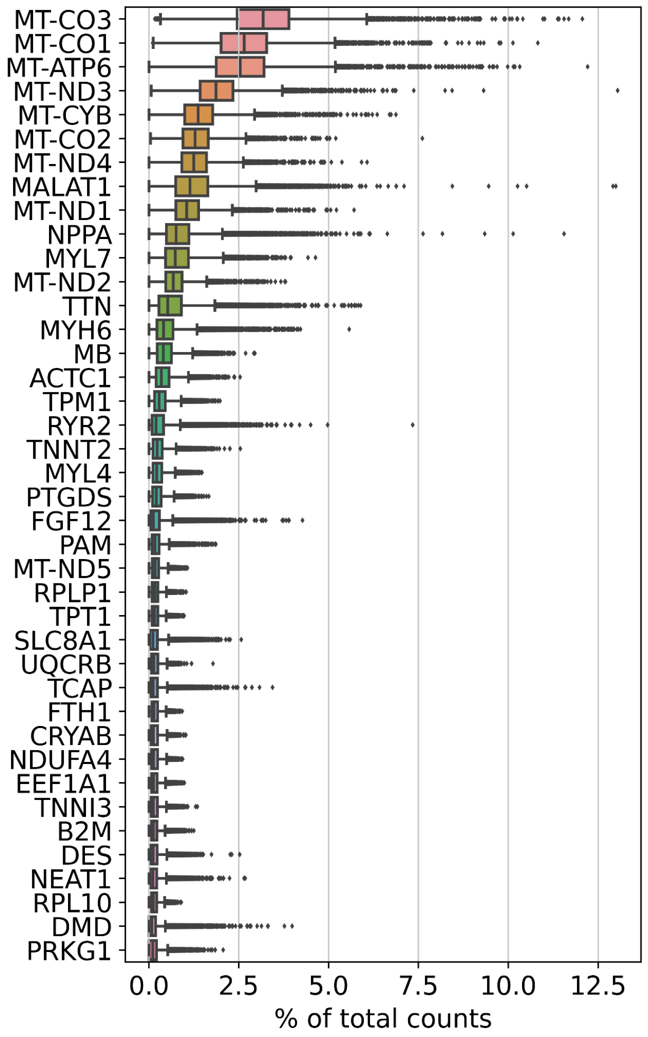

Figure S4. Most abundant genes in an empty droplets-cluster selected by QClus. A high presence of reads stemming from mitochondria and cardiomyocyte marker genes is observed. The sample is from the CAREBANK dataset, sample CB-S00.

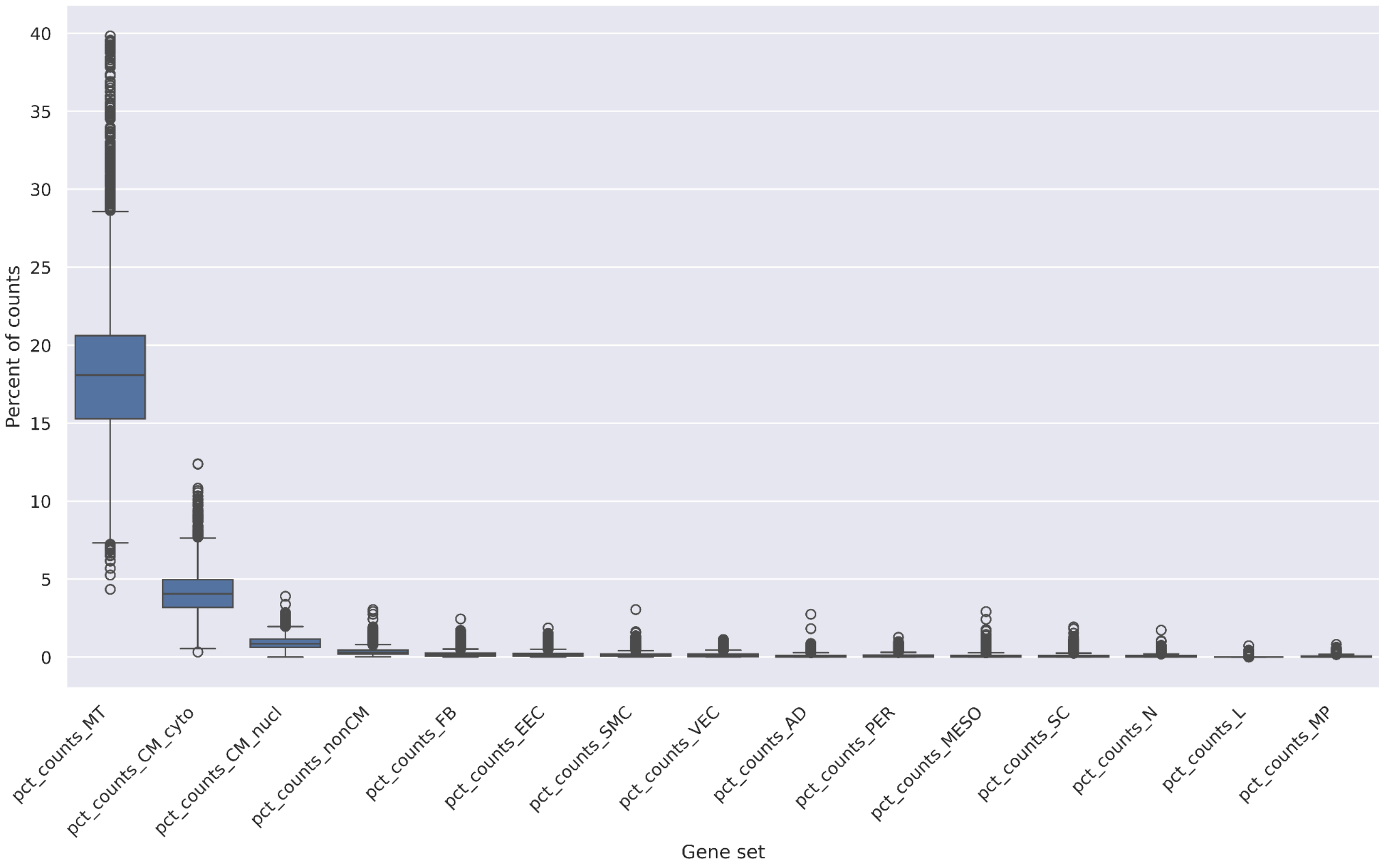

Figure S5. Percentage of counts aligning to the identified marker genes in empty droplets of a single sample. A high fraction of reads align to either mitochondrial genes or genes identified as cytoplasm-enriched cardiomyocyte genes. The sample is from the CAREBANK dataset, sample CB-S00.

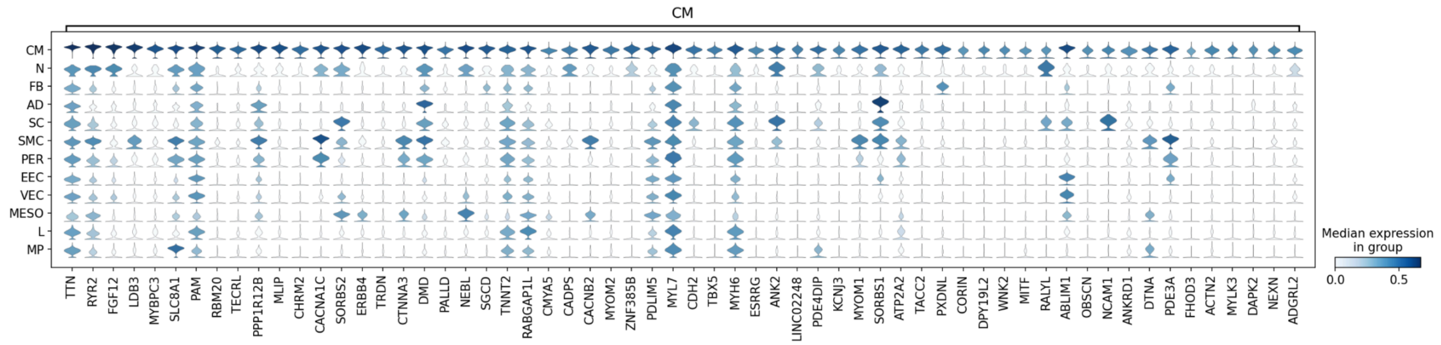

Figure S6. Cardiomyocyte specific gene expression across cell type populations. Cardiomyocyte specific genes can be observed to be either highly specific to only cardiomyocytes (nuclei enriched) or also present to significant degrees in other cell types (cytoplasmic CM genes present in background contamination). The figure is taken from Kuosmanen et al. (1). The sample is from the CAREBANK dataset, sample CB-S00.

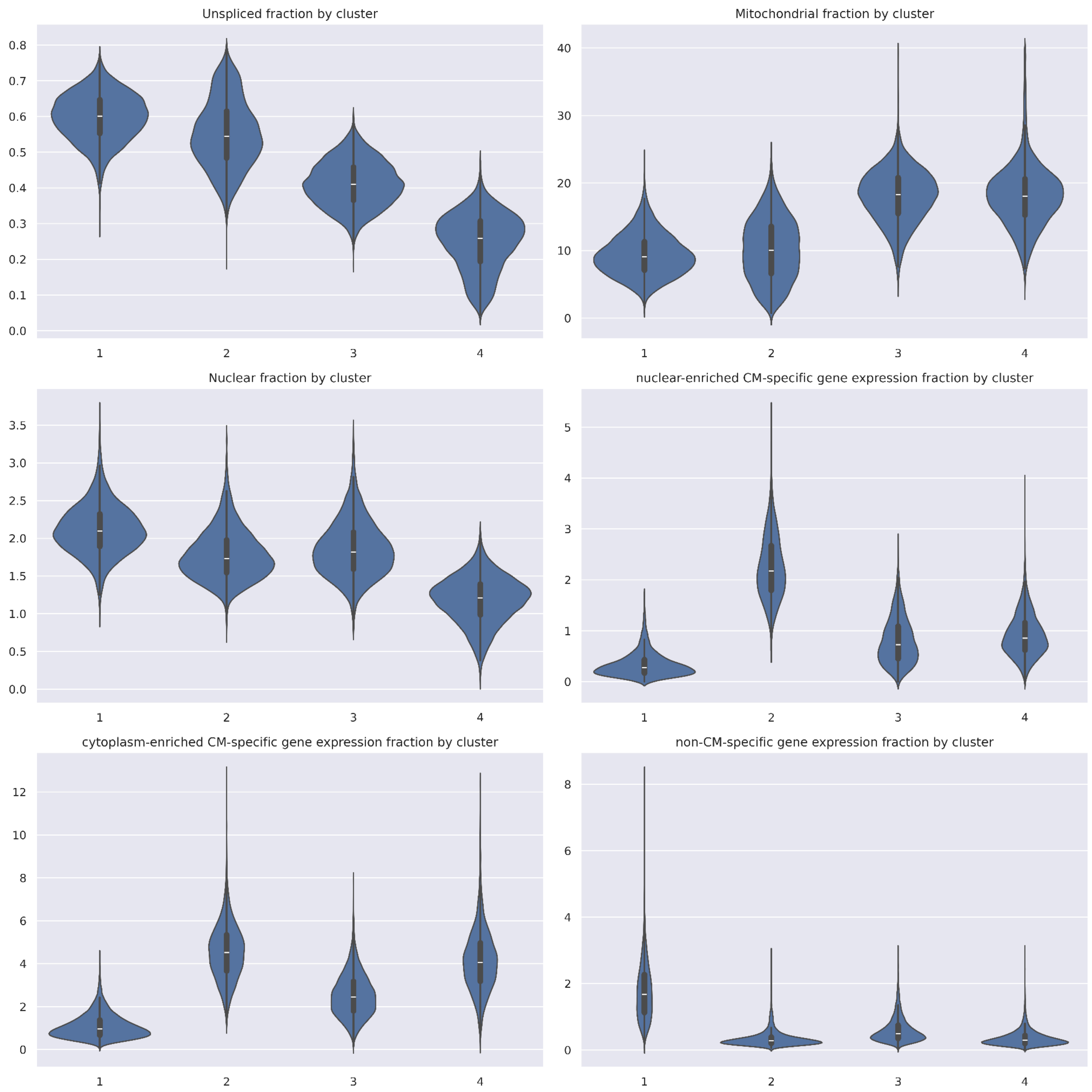

Figure S7. Distribution of clustering metrics across k-means identified clusters. Cluster 1 corresponds to non-cardiomyocyte nuclei. Cluster 2 corresponds to cardiomyocyte clusters. Cluster 3 corresponds to nuclei containing droplets along with some ambient RNA contamination. Cluster 4 corresponds to likely empty droplets. The sample is from the CAREBANK dataset, sample CB-S00.

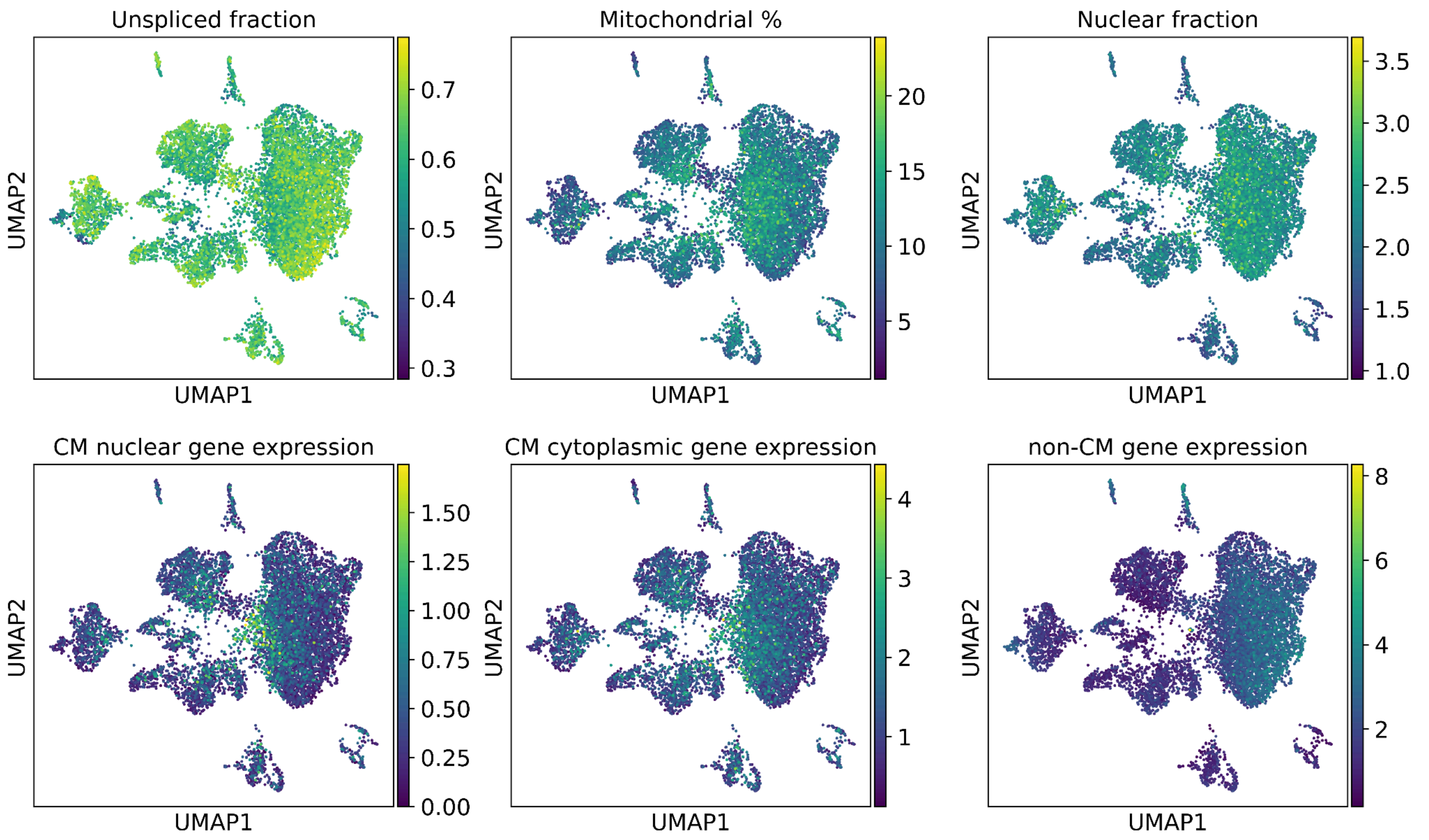

Figure S8. Distribution of clustering/quality metrics across UMAP of cluster 1, which corresponds to non-CM cell type populations. The sample is from the CAREBANK dataset, sample CB-S00.

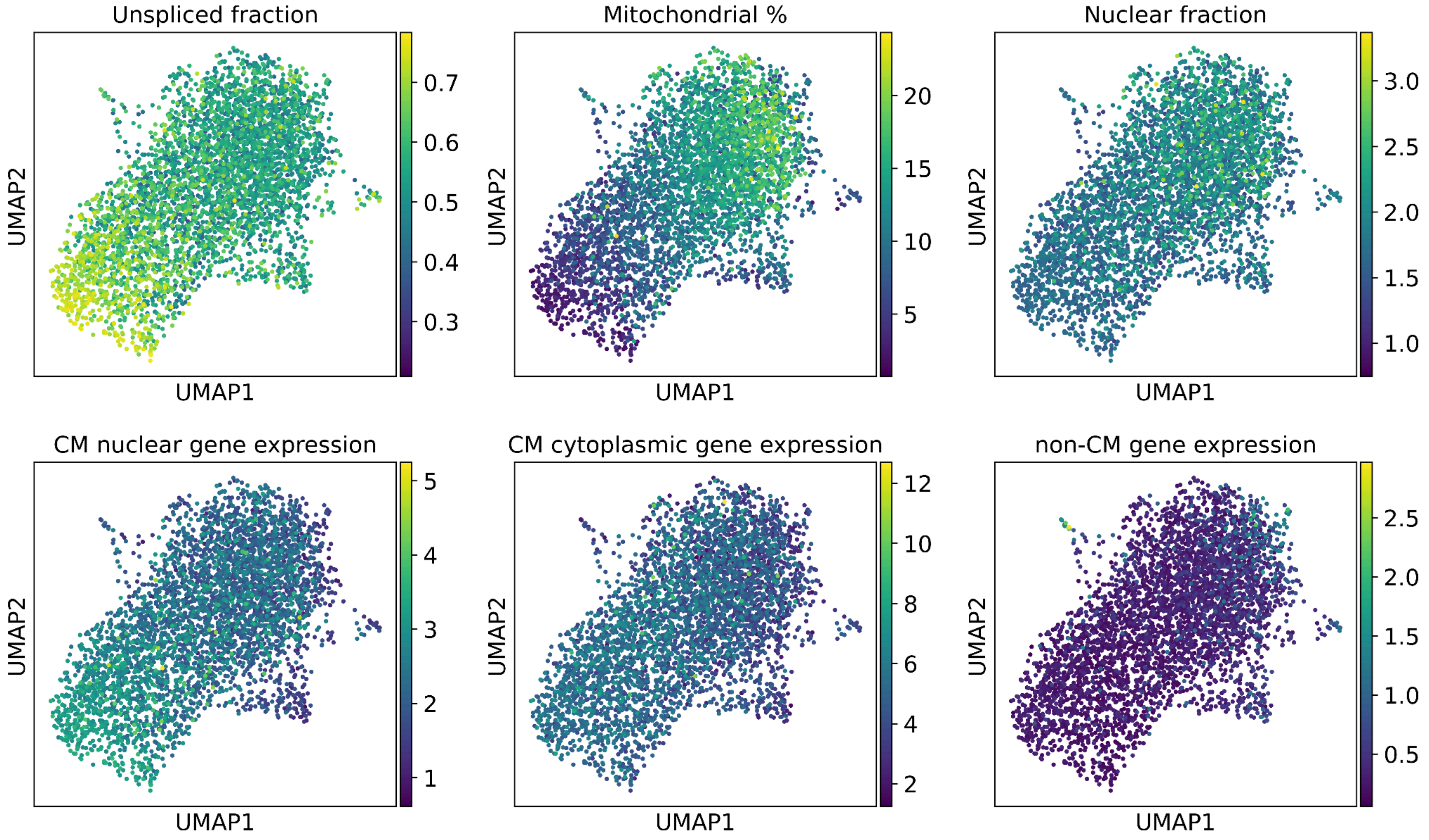

Figure S9. Distribution of clustering/quality metrics across UMAP of cluster 2, which corresponds to cardiomyocytes droplets. The sample is from the CAREBANK dataset, sample CB-S00.

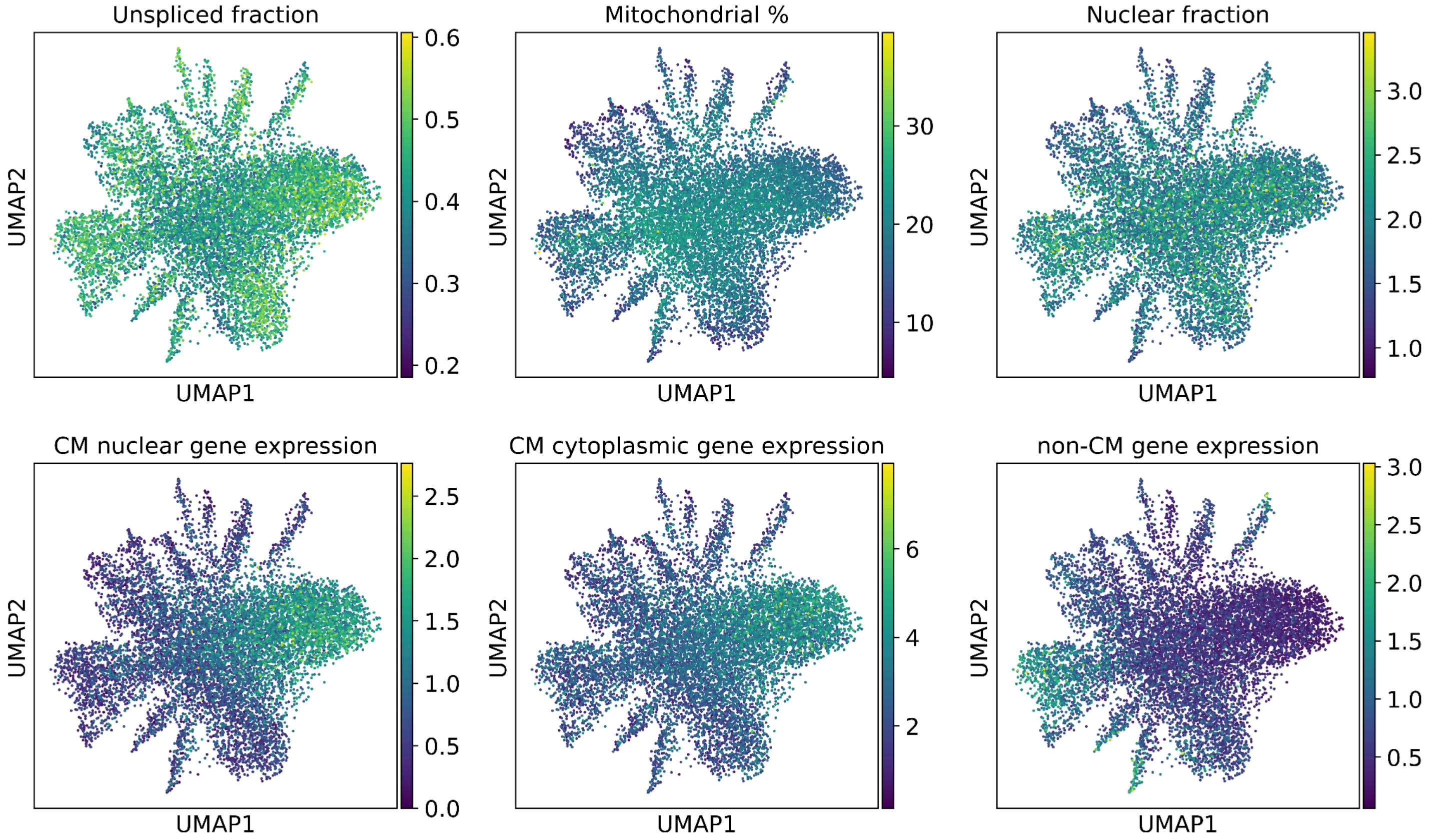

Figure S10. Distribution of clustering/quality metrics across UMAP of cluster 3, which corresponds to highly contaminated droplets. The sample is from the CAREBANK dataset, sample CB-S00.

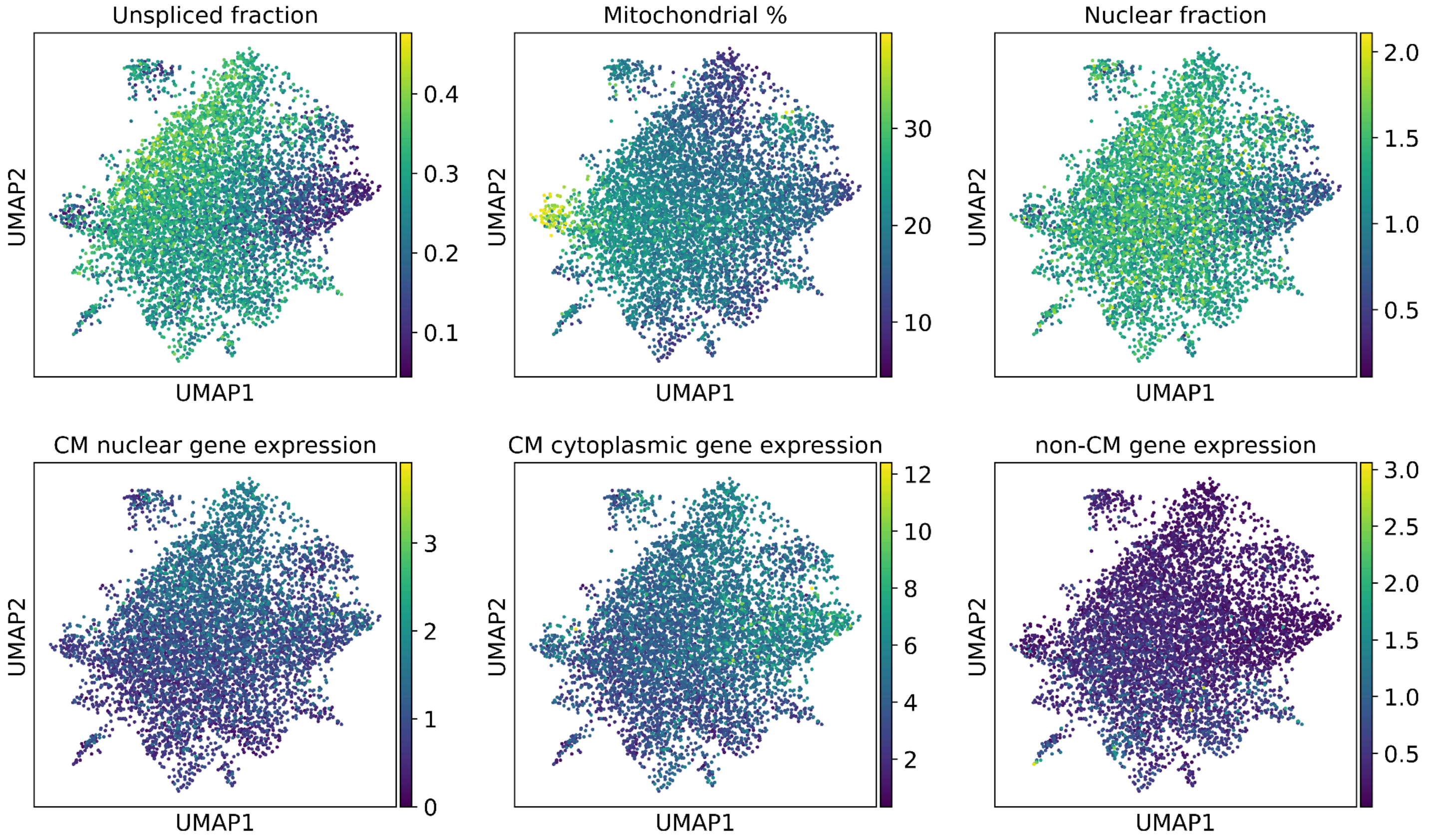

Figure S11. Distribution of clustering/quality metrics across UMAP of cluster 4, which corresponds to empty droplets. The sample is from the CAREBANK dataset, sample CB-S00.

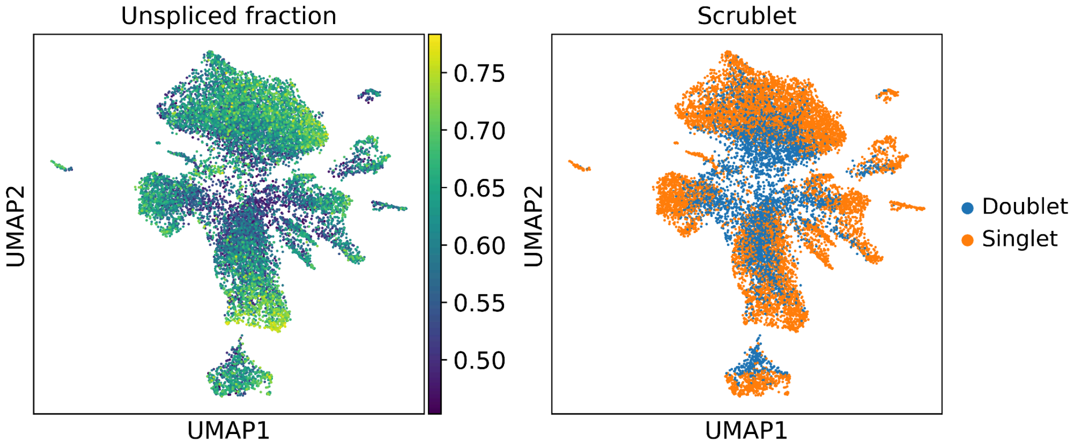

Figure S12. Identified doublets display lower unspliced fraction than non-doublets. The sample is from the CAREBANK dataset, sample CB-S00.

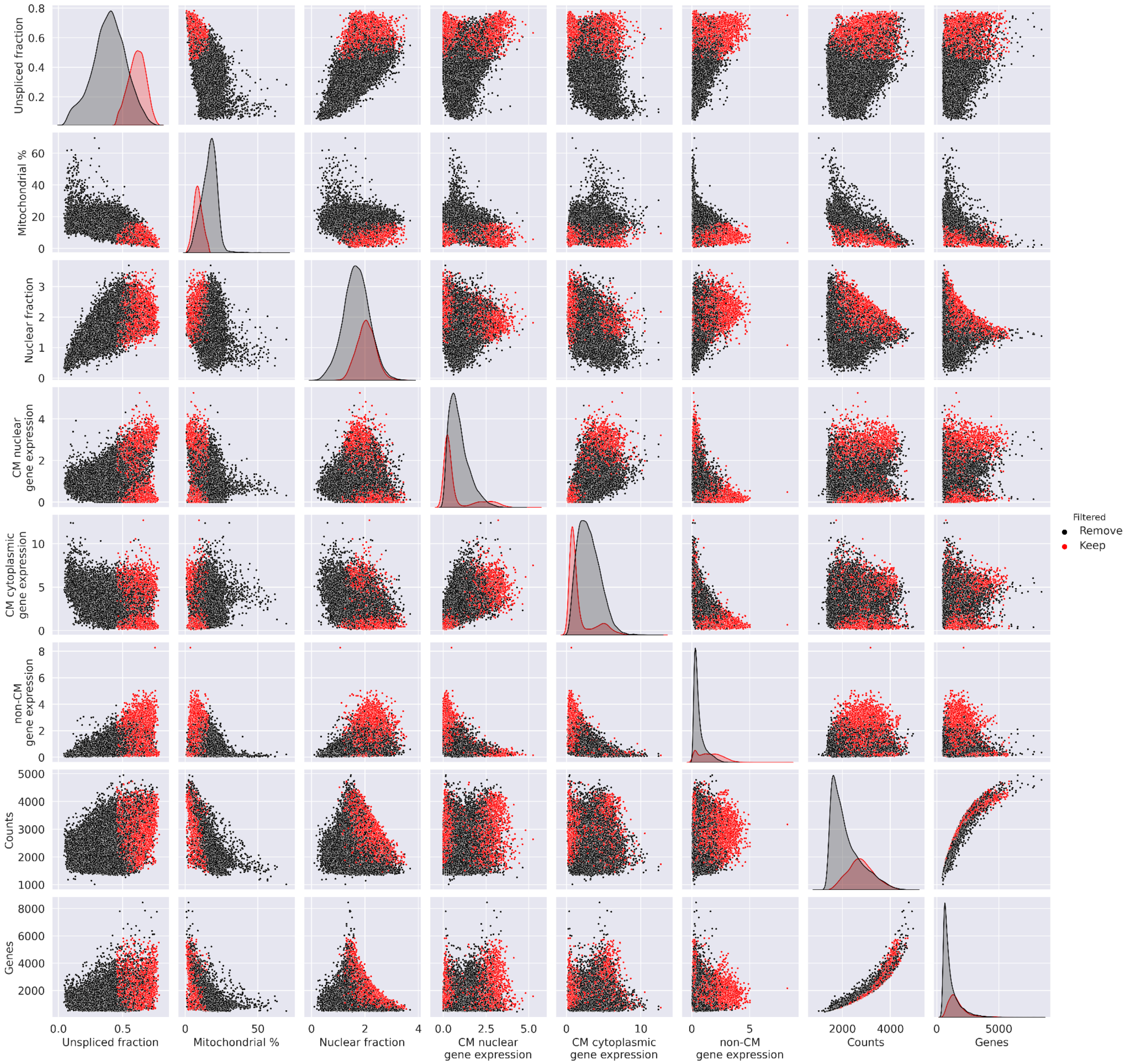

Figure S13. Pairplot of quality metrics colored by whether or not a droplet is kept or removed. Every metric shows overlapping distributions of kept and removed nuclei. The sample is from the CAREBANK dataset, sample CB-S00.

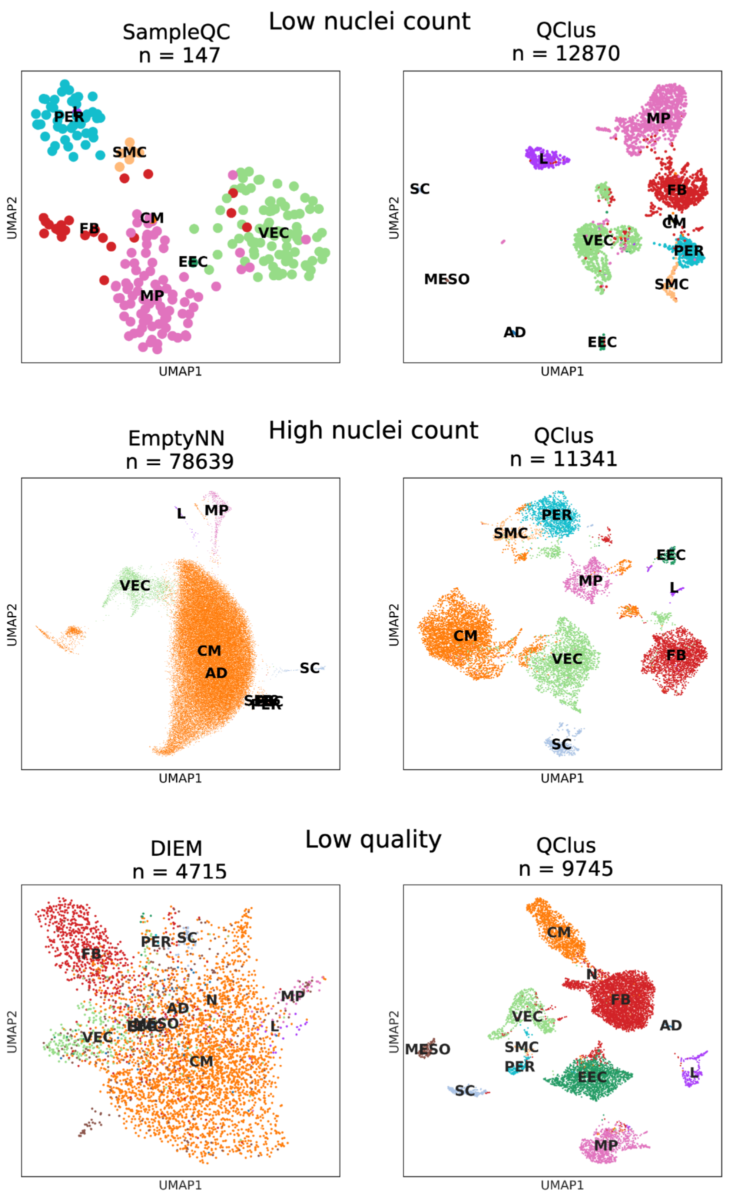

Figure S14. Examples of processing failures in other droplet filtering methods (left column) vs. successful processing with QClus (right column) for three heart example samples. The three samples are: Koenig et al., sample TWCM-13-17 (top row), Hill et al., sample Hi_WU198LV_rep1 (middle row), CAREBANK sample CB-A04 (bottom row).

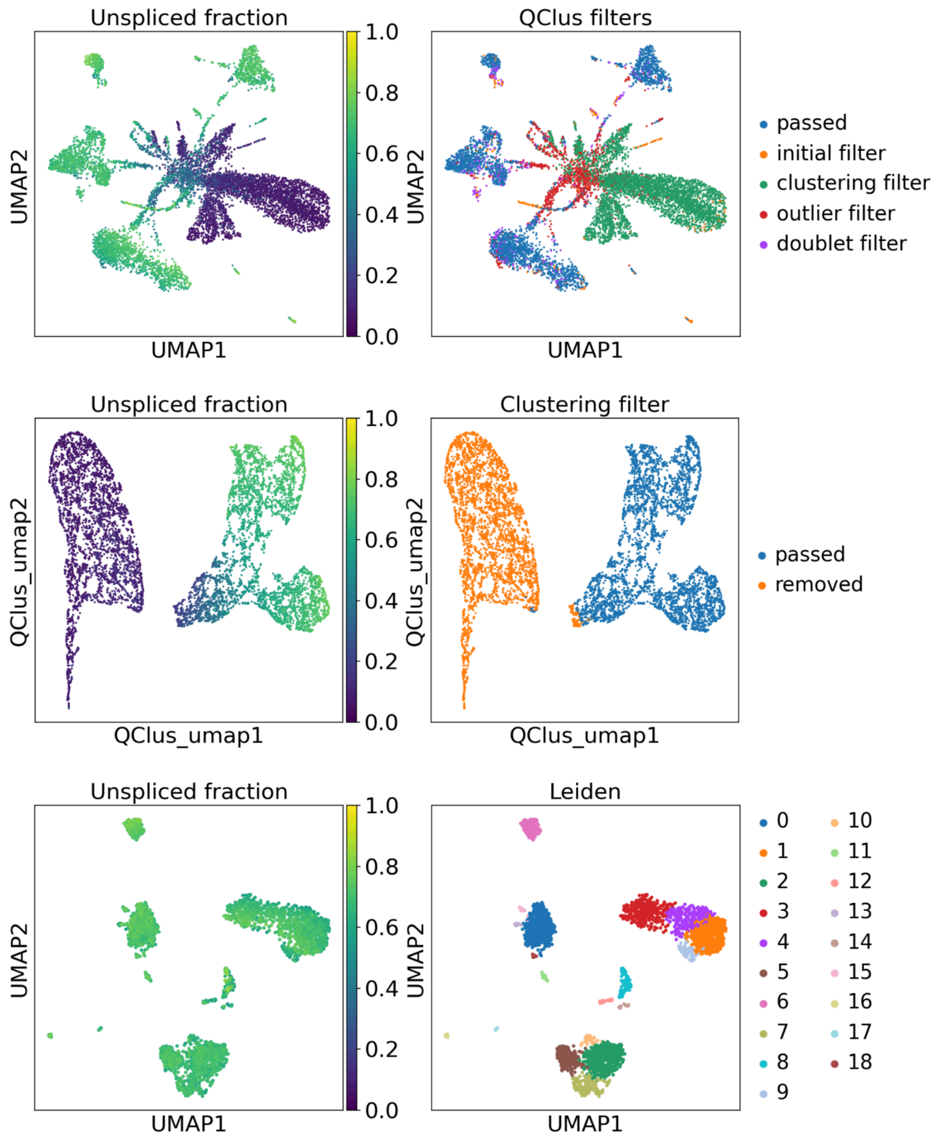

Figure S15. Evaluation of QClus' performance on a low quality brain sample (SAMN37485430) (2). The first row shows a star-shaped pattern with one very low quality branch and a very low quality center (defined by low unspliced reads fraction). When plotting this on a UMAP of the clustered quality metrics, this is visible as a large cluster with near-zero unspliced fraction. This is a common pattern observed in low quality samples.

References

1. Linna-Kuosmanen,S., Schmauch,E., Galani,K., Boix,C.A., Hou,L., Örd,T., Toropainen,A., Stolze,L.K., Meibalan,E., Mantero,J.C., *et al.* (2021) Single-cell dissection of live human hearts in ischemic heart disease and heart failure reveals cell-type-specific driver genes and pathways. 10.1101/2021.06.23.449672.
2. Martirosyan,A., Ansari,R., Pestana,F., Hebestreit,K., Gasparyan,H., Aleksanyan,R., Hnatova,S., Poovathingal,S., Marneffe,C., Thal,D.R., *et al.* (2024) Unravelling cell type-specific responses to Parkinson's Disease at single cell resolution. *Mol. Neurodegener.*, **19**, 7.
