# Supplementary material for: QClus: a droplet filtering algorithm for enhanced snRNA-seq data quality in challenging samples
Source: Nucleic Acids Res. 2024 Dec 5;53(1):gkae1145. doi: 10.1093/nar/gkae1145 (PMC11724311; doi:10.1093/nar/gkae1145)
Supplement: gkae1145_Supplemental_Files [file gkae1145_supplemental_files.zip › Supplementary data table legends.pdf]

**Supplementary Table 1.** Settings used for threshold values in the benchmarking. Details of how these were applied can be found in the methods section.

**Supplementary Table 2.** Gene sets used to calculate non-CM cell type specific quality metrics.

**Supplementary Table 3.** Mean values for the clustering metrics used by QClus across all samples from six datasets. Values are calculated prior to any filtering. Each sample is one datapoint.

**Supplementary Table 4.** Benchmarking results for each processing of all samples. All values are thus calculated after cell filtering. We report the number of nuclei kept, various quality metrics, as well as cell type composition. Empty lines reflect the method failing during the run for that sample (thus removed from the benchmarking of that method).

**Supplementary Table 5.** Scaled results for unspliced fraction, mitochondrial percentage, total counts, and number of genes. Scaling of each metric is performed at the sample level. For details of this scaling, see methods. Empty lines reflect the method failing during the run for that sample (thus removed from the benchmarking of that method).

**Supplementary Table 6.** Number of samples that failed the three quality control criteria across the eight methods. Samples that did not run successfully are annotated under “missing sample”.

**Supplementary Table 7.** Brain dataset processing results. Each sample was processed using QClus. For details of how QClus was applied to the brain dataset, see methods.
